# Impact of COVID-19 on medical education in different income countries: a scoping review of the literature

**DOI:** 10.1080/10872981.2022.2040192

**Published:** 2022-03-22

**Authors:** Niamh Connolly, Mohamed Elhassan Abdalla

**Affiliations:** School of Medicine, University of Limerick, Limerick, Ireland

**Keywords:** COVID-19, coronavirus, medical, education, students, teachers, technology, high-income, middle-income, low-income

## Abstract

The COVID-19 pandemic has disrupted medical education worldwide. Universities were forced to rapidly adapt to the evolving situation and develop methods of delivering curricula and assessments online. The purpose of this scoping review was to assess the impact of COVID-19 on medical education and investigate how this effect varies in different income countries. The methodology adhered to PRISMA (Preferred Reporting Items for Systematic Reviews and Meta-Analyses) extension for scoping reviews. Key terms were searched in six electronic databases. Inclusion criteria included studies describing the effect of COVID-19 on undergraduate medical education in university and clinical settings, studies published post 1 December 2019 and studies published in English. A modified Johanna Briggs Institute data charting tool was used to extract data concerning study characteristics and outcomes. The initial search returned 298 articles. Following duplicate removal and article screening, 33 studies were included. The literature indicated that the pandemic had a negative effect on medical student education worldwide, in both high-income countries (HICs) and low- and middle-income countries (LMICs). A range of factors impacted students and educators, including new curriculum and assessment design, reduced patient contact, use of new technology and lack of infrastructure. However, LMICs encountered more arduous barriers such as lack of access to information technology infrastructure and support from national governments. COVID-19 has impeded medical education worldwide. Future research is needed to address barriers to providing medical education during a pandemic. LMICs need particular support as they have fewer resources and face greater challenges regarding this matter.

## Introduction

The coronavirus pandemic has caused countries worldwide to implement emergency lockdown strategies. This has disrupted medical student education in both university and clinical settings. Universities were forced to cancel on-campus teaching and many hospital placements were suspended or restricted in an effort to halt the spread of COVID-19. A study showed that 90% of medical schools in the USA abruptly ceased on-campus teaching and clinical placements and began online teaching in response to COVID-19 [1]. In Africa, between March and July 2020, 44 out of 54 countries temporarily closed all universities including medical schools [2]. These changes have resulted in face-to-face teaching and practical tutorials being replaced with online learning for medical students. However, virtual teaching methods are of limited value in medical education due to the practical and clinical-based elements of the course [3]. A survey performed on medical students from six universities in the USA demonstrated that 74.7% of this population believed that COVID-19 had significantly disrupted their education [4]. Another study of final year medical students in the UK revealed 59.3% of students either strongly agreed or agreed that they felt unprepared for starting their careers as doctors due to the impact of COVID-19 on their education [5].

COVID-19 is a relatively recent phenomenon, and its effect on medical education is constantly evolving. Universities have been forced to adapt their curricula and examinations to facilitate social distancing measures. The use of technology and online learning has become a core component of medical teaching throughout the world as a result of the pandemic. Medical education and COVID-19 are global issues. However, a recent study demonstrated that the majority of research into this topic thus far has used information from high-income countries (HICs) rather than low- and middle-income countries (LMICs). This study also found that LMICs encounter significantly more arduous challenges in implementing changes in their medical schools in response to the pandemic compared to HICs. Some of these challenges include lack of infrastructure, limited financial resources and lack of trained personnel [6]. A global survey undertaken by the International Association of Universities, assessing the effect of the COVID-19 pandemic on colleges and universities, found that 24% of universities in Africa reported that teaching had been suspended or cancelled with no alternative online platform provided. This compared with 3% of universities in Europe being affected in this way. Only 29% of African teaching faculties could quickly adapt to online teaching compared to 85% of universities in Europe [7]. This indicates an evident discrepancy in the impact of COVID-19 on medical education in different income countries throughout the world.

COVID-19 and its effect on medical education is a new and diverse topic, and therefore, a scoping review was judged as the most effective method to map and evaluate current evidence. Scoping reviews are used to address broad subjects, which have not yet been reviewed comprehensively, and they are often precursors to systematic reviews [8]. They allow for the inclusion of various types of literature, methodologies and data analysis. The purpose of this scoping review was to investigate the different effects of COVID-19 on medical education, which have been reported thus far in the literature. In addition, it aims to assess the way in which this impact differs between different income countries. This scoping review will allow available research to be mapped, results to be summarised and research gaps to be identified. It will also provide direction for future research regarding this subject.

## Methods

This scoping review comprised of a systematic search, research review and descriptive analysis of available literature. This review adhered to the PRISMA (Preferred Reporting Items for Systematic Reviews and Meta-Analyses) extension for scoping reviews [9]. Arksey and O’ Malley’s methodological framework was used [10]. The five steps of this framework include: (i) Identifying the Research Question; (ii) Identifying Relevant Studies; (iii) Study Selection; (iv) Charting the Data and (v) Collating, Summarising and Reporting the Results.

### Identifying the research question

What is the impact of COVID-19 on medical education, and how does this impact differ in different income countries? To allow this research question to be answered, HICs and LMICs had to be clearly identified. This was done using the World Bank Group classification system [11]. This system classifies countries into four groups based on their gross national income (GNI) per capita: high, upper-middle, lower-middle and low. These classifications are outlined in Table 1. For the purpose of this study, upper-middle, lower-middle and low-income countries are classified as LMICs, while high-income countries are categorised as HICs according to the World Bank Group system.

**Table 1.** World Bank Group country classifications by income levels for 2021–2022

Group	Income (GNI per capita in current US dollars, using Atlas method exchange rates)
Low-income	<1045
Low-middle-income	1046–4095
Upper-middle-income	4096–12,695
High-income	>12,695

Source: World Bank Group, 2021.

This research question was considered to be sufficiently broad to capture information relating to this topic. Some factors to be considered within this research question included effects of COVID-19 on curricula and assessments in medical schools, use of technology and infrastructure and the impact the pandemic has had on medical students and educators.

### Identifying relevant studies

Six databases were selected and searched during June 2021. These databases were CINAHL, Cochrane, EMBASE, MEDLINE, Scopus and Web of Science. Search terms were grouped, and Boolean operators were used to yield relevant results. The following key terms were searched as follows: (COVID-19 OR coronavirus) AND (impact OR effect) AND (medicine OR medical) AND (education OR training OR students OR educators OR teachers OR university) AND (high-income countries OR low-income countries OR developed OR developing).

For the purpose of this review, medical students were defined as undergraduate students who had not yet received their primary qualification as a doctor. Inclusion and exclusion criteria were applied to the search strategy. The following inclusion criteria were used: studies describing the effect of COVID-19 on undergraduate medical education in university and clinical settings, studies published post 1 December 2019 when COVID-19 was first recorded and studies published in the English language. The following exclusion criteria were used: studies that described the impact of COVID-19 on the education of post-graduate medical professionals or other healthcare professionals, studies published before 1 December 2019 and studies not published in the English language.

### Study selection

The search methods and criteria outlined above were used. Mendeley v1.19.4 (Elsevier) was used to reflect origin of sources, and duplicate references were removed. The remaining article titles were screened manually by the author to evaluate their capacity to answer the research question. Those deemed not eligible were eliminated from the review. The abstracts of articles with relevant titles were screened and if considered applicable, the full article was reviewed. Only articles relevant to the aims of the study and which met the inclusion criteria were included, while those which did not meet the requisitions were excluded from the review. This selection process was reviewed and verified by the second author.

### Charting the data

A data-charting form was created using Microsoft Excel. This data-charting form was adapted from the Johanna Briggs Institute (JBI) data extraction tool [12]. Data was extracted independently from the articles and categorised according to the following headings: Author(s), Title, Year of Publication, Country of Study, World Bank Group Country Classification by Income Level, Design of Study, Number of Participants, Aim(s) of Study, Data Collection Duration and Key Findings.

### Collating, summarising and reporting the results

A narrative analysis of the heterogenous studies was completed. Gaps in knowledge were identified, and the existing data was assessed to investigate how these gaps could be addressed in future research. This is a scoping review, and therefore quality assessment of the outcome measures was not a primary objective.

## Results

### Mapping the results

**Figure 1.**
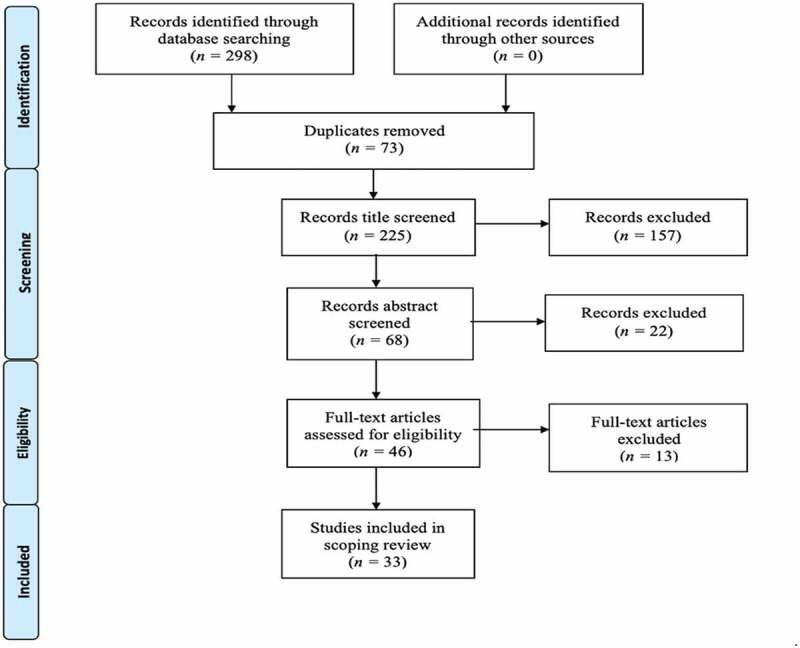
PRISMA flowchart

The initial search identified *n* = 298 articles. Following a duplicate screen, *n* = 73 articles were removed. Article titles were then screened according to PRISMA guidelines, and all titles that were not applicable to the research question (*n* = 157) were eliminated. The remaining *n* = 68 abstracts were reviewed, and their relevance to the research question was assessed. This resulted in a further *n* = 22 abstracts being eliminated and *n* = 46 full articles being reviewed to deem their eligibility to be used in this study. A further *n* = 13 articles were eliminated after this review. This was largely due to the studies not fulfilling inclusion criteria. Therefore, the final number of articles used in the scoping review was *n* = 33. The PRISMA flowchart demonstrates this search strategy and study selection (see Figure 1). The included studies were summarised in Table 2.

**Table 2.** **Summary Table of Included Studies** x = Information not available or not applicable to study

Author(s)	Title	Year of Publication	Country of Study	World Bank Income Classification of Country	Design of Study	Number of Participants	Aim(s) of Study	Data Collection Duration	Key Findings
Bączek *et al* [13]	Students’ perception of online learning during the COVID-19 pandemic	2021	Poland	High-income	Survey study	804participants	To analyse medical students’ perception of online learning during the COVID-19 pandemic.	4 weeks and 3 days	Main advantages of online learning: ability to stay at home (69%), continuous access to online materials (69%), learning at own pace (64%) and comfortable surroundings (54%). Main disadvantages of online learning: lack of interaction with patients (70%) and technical problems with IT equipment (54%). No statistical difference between face-to-face and online learning in relation to increasing knowledge. E-learning is less effective than face-to-face learning in increasing skills and social competencies. 73% of students found online learning enjoyable.
Choi *et al* [5]	The Impact of the COVID-19 Pandemic on Final Year Medical Students in the United Kingdom: A National Survey	2020	UK	High-income	Survey study	444 participants	To identify the impact of COVID-19 on final year medical students’ examinations and placements in the UK and how it might impact on their confidence and preparedness going into their first year of foundation training.	x	38.4% of participants had their final OSCEs cancelled (43% had already completed these exams before restrictions were imposed). 18.6% had simulated patients or OSCE stations requiring patient contact cancelled. 55.9% reported no change to written examinations while 26.8% completed them online remotely. 9.8% reported cancellations. Students’ confidence for starting foundation training was significantly affected by the impact of COVID-19 on student assistantships only.
Compton *et al* [14]	Medical students’ preference for returning to the clinical setting during the COVID-19 pandemic	2020	Singapore	High-income	Survey study	179 participants	To assess medical students’ preference for re-entering the clinical setting during the COVID-19 pandemic and to explore personal and environmental characteristics associated with that preference.	9 days	One third of participants preferred not to return to the clinical setting due to COVID-19. Two-thirds of participants wanted to return to clinical placement. Reasons for this included improving clinical capacity.
De Ponti *et al* [15]	Pre-graduation medical training including virtual reality during COVID-19 pandemic: a report on students’ perception	2020	Italy	High-income	Survey study	115 participants	To assess medical students’ perception of online training including simulated clinical scenarios during the COVID-19 pandemic.	x	90% of participants evaluated virtual reality training positively. 77% thought virtual reality training was realistic for initial clinical assessment. 94% thought virtual reality training was realistic for diagnostic activity. 28% found online access difficult due to technical issues.
Dost *et al* [16]	Perceptions of medical students towards online teaching during the COVID-19 pandemic: a national cross-sectional survey of 2721 UK medical students	2020	UK	High-income	Cross-sectional, online national survey study	2721 participants	To investigate perceptions of medical students on the role of online teaching in facilitating medical education during the COVID-19 pandemic.	1 week	Overall, students did not find online teaching to be engaging or enjoyable. They also did not find it as effective as face-to-face teaching. 76% felt online teaching did not successfully replace clinical teaching via direct patient contact. 82% felt they could not learn practical clinical skills through online teaching. Main advantages of online learning were saving time on travelling (19.82%), flexibility (19.52%) and allowing students to learn at their own pace (18.63%). Main barriers to online learning were family distraction (26.76%) and poor internet connection (21.53%).
Elzainy *et al* [17]	Experience of e-learning and online assessment during the COVID-19 pandemic at the College of Medicine, Qassim University	2020	Saudi Arabia	High-income	Cross-sectional study	250 participants (pre-clinical phase)	To explore the impact of e-learning and assessment on the performance of students and faculty, and the challenges to their sustainability.	65 days	78% of students either strongly agreed or agreed that e-learning compensated for face-to-face teaching. 40% either strongly agreed or agreed that staff had enough experience in e-learning requirements while 30% either strongly disagreed or disagreed on the same issue. 68% either strongly agreed or agreed that interaction during the online sessions was satisfactory. 60% either strongly agreed or agreed that online assessment is effective to test knowledge.
Franklin *et al* [18]	How the Covid-19 Pandemic Impacted Medical Education During the Last Year of Medical School: A Class Survey	2021	USA	High-income	Cross-sectional, mixed method survey	63 participants	To get a better understanding of how the COVID-19 pandemic impacted fourth-year medical students’ learning, after a complete changeover from in-person to remote learning.	6 days	57% indicated that their sub-speciality was impacted by COVID-19. 35% of students were satisfied with use of their e-learning platform. Students did not find tele-education and e-learning as effective as traditional medical education.
Haley *et al* [19]	The Negative Impact of COVID-19 on Medical Education amongst Medical Students Interested in Plastic Surgery: A Cross-sectional Survey Study	2021	USA	High-income	Cross-sectional study	130 participants	To investigate how medical students interested in plastic surgery were impacted by COVID-19-associated curriculum changes.	1 month	91% of students attended schools that offered online courses and 46% still had in-person rotations with restricted schedules. 80% believed that the pandemic had a negative impact on their medical education. 94% felt that their medical schools were ‘’fairly supportive’’ of their psychosocial wellbeing during COVID-19.
Harries *et al* [4]	Effects of the COVID-19 pandemic on medical students: a multicenter quantitative study	2021	USA	High-income	Cross-sectional survey study	741 participants	To investigate the educational and psychological effects of the pandemic on US medical students and their reactions to the Association of American Medical Colleges (AAMC) recommendation to pause all student clinical rotations with in-patient care, in order to inform medical education policy.	35 days	74.7% of students felt the pandemic had significantly disrupted their medical education. 61.3% believed they should continue with their normal clinical rotations during the pandemic. 61.4% felt COVID-19 had disrupted their skill development to prepare for residency. 72.7% felt medical schools were doing their best to help students adjust.
Kim *et al* [20]	How medical education survives and evolves during COVID-19: Our experience and future direction	2020	South Korea	High-income	Survey study	362 participants	(1) To assess the experience of running a medical school curriculum during COVID-19 by moving all classes online and minimising face-to-face contact. (2) To investigate student satisfaction, problems, achievements and perspectives on the future in relation to COVID-19.	x	Students were generally satisfied with the online course. Students had a higher preference for online lectures (63%) compared to offline lectures (29%), while professors preferred offline lectures (77.3%) over online lectures (31.9%). 84.3% of students wanted to maintain online courses after the pandemic. Among them, 45.5% wanted to combine offline and online classes while 38.8% wanted most lectures to be maintained online. 47.7% of professors wanted to maintain online lectures while 52.3% wanted to return to offline lectures.
Loda *et al* [21]	Medical education in times of COVID-19: German students’ expectations – A cross-sectional study	2020	Germany	High-income	Cross-sectional survey study	372 participants	To investigate the teaching- and learning-related stressors and expectations of medical students in Germany during the COVID-19 pandemic.	3 weeks	Students reported being afraid of losing a semester due to COVID-19. Students reported general uncertainty and scarcity of information as the greatest stressors, followed by worries regarding examinations, practical years and training terms abroad. 71% wanted their teachers to be willing to enhance digital competencies and 66.4% expected teachers to be lenient about examinations.
Motte-Signoret *et al* [22]	Perception of medical education by learners and teachers during the COVID-19 pandemic: a cross-sectional survey of online teaching	2021	France	High-income	Cross-sectional survey study	80 participants	To investigate how online teaching was perceived by both teachers and learners during the COVID-19 pandemic, in order to help determine how to adapt curricula over the coming years.	2 months	85.5% of students agreed that online teaching was an appropriate teaching method during the pandemic. Approximately 70% of students and 69% of teachers felt that they have not received or provided training of an equivalent level and quality as expected. Approximately 15% of students and 38.5% of teachers felt that an online curriculum should continue after the pandemic.
Nishimura *et al* [23]	Impact of the COVID-19 Pandemic on the Psychological Distress of Medical Students in Japan: Cross-sectional Survey Study	2021	Japan	High-income	Cross-sectional survey study	473 participants	To provide details on how medical students have been affected by the pandemic in Japan.	1 week	29.8% of students were concerned about the shift to online teaching, mostly because they thought online teaching would be ineffective compared to face-to-face teaching. 25.6% were concerned about effects on their future career. 49.7% were concerned about their relationships with teachers. Those who had concerns had higher odds of having generalised anxiety and being depressed.
Paton *et al* [24]	Medical Student Experience During the COVID-19 Pandemic: A Qualitative Study	2020	USA	High-income	Qualitative, descriptive study	11 participants	To learn the perspectives of medical students at a single institution regarding the impact of COVID-19 on their education, identify common challenges that they faced, and gather recommendations to approach medical education during similar national or global crises in the future.	10 days	Most students reported that the interruption in clinical rotations negatively affected their education. Reasons for this negative effect included loss of clinical training, access to preceptors, career planning and missing critical clinical electives and opportunities. Students reported they were learning about the form and function of the healthcare system which could not be taught in a classroom setting.
Shahrvini *et al* [25]	Pre-clinical remote undergraduate medical education during the COVID-19 pandemic: a survey study	2021	USA	High-income	Survey study	104 participants	To better understand the effects of the complete transition to remote learning during the COVID-19 pandemic on pre-clinical students.	x	Overall, students felt that online learning had negatively affected teaching quality and their ability to participate. Only 25.5% still felt connected to the medical school or classmates. Anxiety and isolation were reported. 56.7% felt their preparation for exams was negatively affected. 43.3% felt unprepared to begin clerkships.
Torda *et al* [26]	How COVID-19 has pushed us into a medical education revolution	2020	Australia	High-income	Literature review	x	To highlight the way in which COVID-19 has changed medical education.	x	The transition from face-to-face to online teaching was relatively easy for lectures and small group teaching. However, it was more difficult for practical science classes and clinical skills teaching. Senior year medical students were paired with a junior medical officer so they received training with clinical teams and could alleviate pressure on junior medical staff. Campus-based examinations were put online. OSCEs were not possible so other online simulated options were investigated.
Rajab *et al* [27]	Challenges to Online Medical Education During the COVID-19 Pandemic	2020	Saudi Arabia	High-income	Cross-sectional study	177 participants	To analyse the impact of the COVID-19 pandemic on online education at the College of Medicine of Alfaisal University in Riyadh, Saudi Arabia.	March-April 2020	37.4% of medical students and 57.9% of the faculty reported having little or no experience with online teaching before the pandemic. Challenges to online learning include issues regarding in-person communication, assessment, technology tools, experience in online learning, pandemic-related anxiety and stress, learning curve, time management, students’ evaluations of faculty and technophobia.
Vatier *et al* [28]	Lessons from the impact of COVID-19 on medical educational continuity and practices	2021	France	High-income	Survey study	698 participants	To determine the impact of the COVID-19 outbreak on French undergraduate medical students from Sorbonne University.	41 days	15% of teachers did not do any of their usual teaching duties, 50% maintained less than half of their classes, and 13% gave all scheduled lessons. 6% of students did not follow any lessons, 36% followed less than half of the course and 21% used all resources available. 55% of medical students regretted not having face-to-face teaching and 70% of teachers had this same regret.
Al-Balas *et al* [29]	Distance learning in clinical medical education amid COVID-19 pandemic in Jordan: current situation, challenges, and perspectives	2020	Jordan	Upper-middle- income	Cross-sectional study	652 participants	To explore the situation of distance e-learning among medical students during their clinical years and to identify possible challenges, limitations, satisfaction and perspectives for this approach to learning.	x	26.77% of students were satisfied with their experience in distance learning, 28.81% felt neutral and 44.42% were dissatisfied with this experience. 55.9% of students reported multiple advantages including time-saving, flexibility and improved interaction with instructors and classmates. 48.3% reported low quality of teaching, 62.1% reported poor interaction with instructors and 69.2% reported internet quality issues.
Alsoufi *et al* [30]	Impact of the COVID-19 pandemic on medical education: Medical students’ knowledge, attitudes, and practices regarding electronic learning	2020	Libya	Upper-middle- income	Cross-sectional survey study	3348 participants	To provide an overview of the situation experienced by medical students during the COVID-19 pandemic and to determine the knowledge, attitudes, and practices of medical students regarding electronic medical education	May-June 2020	97.1% reported suspended lectures and educational programs, while 86% reported suspended clinical training and laboratory skills training. 35.1% and 29% of students reported that they had access to a good or very good internet connection, respectively. 64.7% disagreed that e-learning could be used easily in Libya. 78.3% found it difficult to participate in e-learning due to financial cost.
Aziz *et al* [31]	Impact of Covid-19 on education of undergraduate medical students in Pakistan.	2020	Pakistan	Lower-middle-income	Cross-sectional study	765 participants	To assess the impact of COVID-19 on the education of undergraduate medical students in Pakistan.	1 month	96% of students had online classes but 52.8% reported that these were not effective. 69.7% of students were dissatisfied with e-learning. 91.5% reported that they have lost interest in studies. 88% rejected online assessment as an alternative to traditional examinations.
Baticulon *et al* [32]	Barriers to Online Learning in the Time of COVID-19: A National Survey of Medical Students in the Philippines	2021	Philippines	Lower- middle-income	Cross-sectional study	3870 participants	To identify barriers to online learning from the perspective of medical students in a developing country.	14 days	Only 41% of students were capable of adapting to online learning. 44% reported that their schools were equipped to support online teaching. Common barriers to online learning included difficulty adjusting to a new learning style, having to perform other responsibilities at home, poor communication from educators, lack of physical space and lack of sufficient internet connection.
Daroedono *et al* [33]	The impact of COVID-19 on medical education: our students perception on the practice of long distance learning	2020	Indonesia	Lower- middle- income	Cross-sectional survey study	545 participants	To measure the impact of COVID-19 on medical education by asking the students’ perception on long distance learning delivered during this pandemic.	2 weeks	The greatest perceived benefits of distance learning by medical students were: location flexibility (87.9%), time flexibility (76.5%), low cost (65.7%) and no specific preparation needed (57.6%). The greatest perceived disadvantages were: being internet signal dependent (80.2%), costs for additional cellular data (79.1%), lack of understanding (77.8%) and lack of concentration (77.6%).
Liu *et al* [34]	The impact of COVID-19 on medical education: Experiences from one medical university in Taiwan	2021	China	Upper- middle- income	Short communication	x	To present the strategies taken by Taiwanese medical schools to continue in-person medical education on campus when COVID-19 first occurred.	x	Of all strategies introduced in medical universities to combat COVID-19, adjusting classes to a largely online format had the most significant and lasting effect on students. Lack of staff training in using technology, internet instability and lack of motivation and time management skills were all reported disadvantages of online learning. Time flexibility was a reported advantage of online learning.
Nepal *et al* [35]	Students’ Perspective on Online Medical Education Amidst the COVID-19 Pandemic in Nepal	2020	Nepal	Lower- middle- income	Cross-sectional survey study	226 participants	To analyse medical students’ perspective in Nepal on the newly introduced online medical education system due to COVID-19.	5 days	74.3% of students reported that online classes were poorer than traditional teaching 77.8% would choose traditional teaching over online teaching. 55.8% reported that they did not engage with lessons or reading from e-books before or after online classes.
Oladipo *et al* [36]	Challenges with medical education in Nigeria in the COVID-19 era	2020	Nigeria	Lower- middle- income	Survey study	72 participants	To study the impact of the COVID-19 pandemic on medical education in Nigeria.	x	75% of students reported that e-learning platforms were not used in their institutions before the pandemic. Due to mandatory closing of schools, 45% of schools continue to provide medical education via online learning. All private institutions could continue education while only a few public institutions could do this. Transition to e-learning took 4-8 weeks, indicating some students had no medical education for up to approximately 8 weeks.
Rafi *et al* [37]	Concerns and confidences expressed by teaching staff about the shift of medical education to online mode in South India during the COVID 19 pandemic	2020	India	Lower- middle- income	Survey study	51 participants	To investigate the concerns and confidences of medical teaching staff in India regarding the effect of COVID-19 on undergraduate medical education.	1 week	All teachers preferred regular classroom teaching due to better teacher-student interaction. One third of teachers wanted the long-term continuation of online classes. 47% of teachers self-graded the success rate of online classes as 90% or higher and 43% graded it between 71% and 90%.
Shehata *et al* [38]	Medical Education Adaptations Post COVID-19: An Egyptian Reflection	2020	Egypt	Lower- middle- income	2-phase mixed method exploratory study	78 participants	To explore how medical schools in Egypt responded to the COVID-19 pandemic regarding teaching, learning and assessment for undergraduate students.	x	55.1% of faculty evaluated staff level of preparedness for the changes caused by the pandemic as optimum to high. Only 17.9% reported that technology preparedness for this change was high. 67.9% reported that alternative examination methods were used for formative assessment but 75.6% stated that there was an absence of alternative methods for summative assessment.
Tempski *et al* [39]	Medical students’ perceptions and motivations during the COVID-19 pandemic	2021	Brazil	Upper- middle- income	Survey study	10443 participants	To evaluate the motivation of medical students to be part of health teams to help in the COVID-19 pandemic	3 days	39.7%, 29% and 29% of 1^st^ /2^nd^ years, 3^rd^/4^th^ years and 5^th^/6^th^ years respectively, reported that they would prefer to delay their training to fully replace academic activities rather than participating in distance learning. 41.9%, 56.3% and 58.8% of 1^st^ /2^nd^ years, 3^rd^/4^th^ years and 5^th^/6^th^ years respectively, reported that they felt able to study their course through distance learning. 37.2%, 48.7% and 40.3% of 1^st^ /2^nd^ years, 3^rd^/4^th^ years and 5^th^/6^th^ years respectively, reported that they believed that after the pandemic, only practical elements of the course must be resumed.
Thomas *et al* [40]	Survey Among Medical Students During COVID-19 Lockdown: The Online Class Dilemma	2020	India	Lower- middle- income	Cross-sectional survey study	1016 participants	To evaluate the attitudes of, and the factors affecting, medical students attending online classes during lockdown.	2 days	Network issues was the most common reason (85.8%) why participants disliked online learning. Learning at leisure was the most common reason (44.5%) why participants liked online learning. 51.7% of participants did not want to continue online classes after the pandemic. 55% favoured traditional classes compared to online classes.
Tuma *et al* [41]	Students and faculty perception of distance medical education outcomes in resource-constrained system during COVID-19 pandemic. A cross-sectional study	2021	Iraq	Upper- middle- income	Survey study	717 participants	To describe, evaluate and provide pertinent recommendations about interactive distance education in experience and resource-limited medical schools during the COVID-19 pandemic.	x	27% of students and 49% of instructors either agreed or strongly agreed that the expectations and objectives of online learning activities were achieved. 53% of instructors reported that knowledge gain and teaching effectiveness were similar or better than traditional learning. 67% of students reported more difficulties with online learning compared to traditional learning. 67% of students reported fatigue or loss of interest while participating in online learning.
Yu *et al* [42]	Analysis of factors influencing the network teaching effect of college students in a medical school during the COVID-19 epidemic	2021	China	Upper- middle- income	Cross-sectional survey study	7084 participants	To understand the influencing factors of Chinese college students’ satisfaction with online teaching and psychological pressure associated with learning during the coronavirus epidemic.	1 month	Students from urban areas rated higher than those from rural areas in preparation for their classes, access to information on teaching arrangements and teaching methods, answers to questions and satisfaction with teaching results. The psychological pressure on learning in urban area students was lower than that in rural area students. Satisfaction of female students with teachers’ preparation was higher than that of male students. Students who used computers/tablets as learning tools had higher satisfaction than those who used mobile phones as learning tools.
Gismalla *et al* [43]	Medical students’ perception towards E-learning during COVID 19 pandemic in a high burden developing country	2021	Sudan	Low-income	Cross-sectional survey study	358 participants	To assess medical students’ perception towards implementing e-learning during COVID-19 and to highlight e-learning implementation in Sudan as an example of a limited-resource setting.	16 days	64% of medical students believe that e-learning is the best solution for medical education during the pandemic. Factors reported against e-learning implementation in Sudan include: limited internet connectivity (38%), limited technical support (24%), unfamiliarity with e-learning systems (22%) and lack of face-to-face interaction (16%).

### Study characteristics

**Figure 2.**
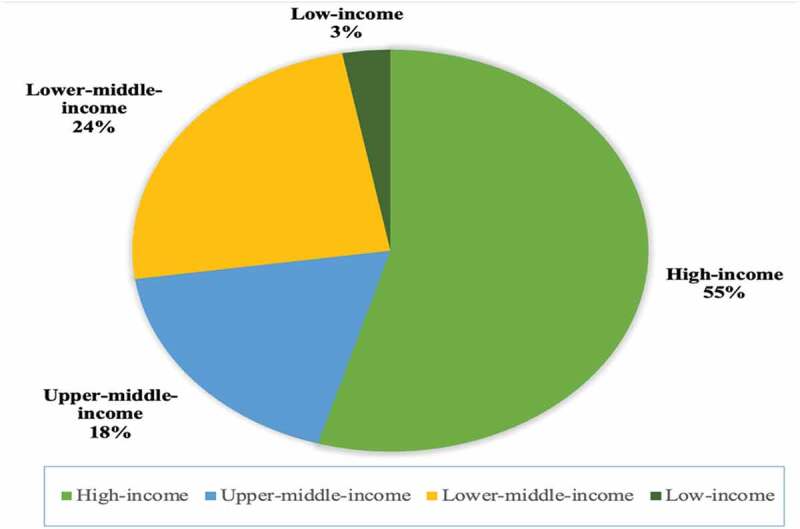
World Bank Group income classification of countries included in review

A time restriction was applied to the search strategy as one of the inclusion criteria was including studies published post 1 December 2019. Therefore, the studies included in this review were published in 2020 and 2021. *n* = 18 of the articles were based on studies from HICs while the remaining *n* = 15 studies were from LMICs (see Figure 2). More specifically, of these 15 studies from LMICs, *n* = 6 were from upper-middle-income countries, *n* = 8 were from lower-middle-income countries and *n* = 1 was from a low-income country. The majority of these studies used survey-based methods to assess student and educator perspectives on the effect of COVID-19 on medical education. The number of participants in the studies varied greatly, ranging from 11 to 10,443 participants.

### Study findings

The findings of the included studies were analysed and reported under the following headings: Student Perspective, Educator Perspective, Technology and Infrastructure and Curriculum and Assessment.

### Student perspective

The overwhelming opinion of students from both HICs and LMICs was that COVID-19 had disrupted their education in several ways. A survey of 2721 medical students from 39 medical schools in the UK showed that overall, students did not find online teaching enjoyable or engaging; 76% of participants reported that they did not feel online teaching was effective at replacing learning through direct patient contact. However, some advantages of online learning were also reported by students, including greater flexibility, time saved on travelling to university and less costs incurred compared to physically attending university [16]. Other studies from both HICs and LMICs reported similar benefits from the transition to online teaching [13,29,33]. A study of clinically rotating medical students from six medical schools in the USA reported that 61.4% of participants felt that the pandemic had disrupted their skill development, which would be necessary for residency preparation. However, 72.7% of participants in this study also felt that their medical institutions were doing their best to help students during this crisis [4]. Loneliness and isolation were common factors reported by students. A study in a Parisian university found that 53% of medical students reported a lack of motivation to study due to feelings of isolation during distance learning [28]. Similar feelings of anxiety and isolation were reported by medical students in the USA in response to online learning [25]. Students in LMICs reported similar disadvantages as those in HICs. A survey of medical students in Pakistan revealed that 75.7% of students had negative perceptions towards e-learning [31]. Another study in Nepal showed that 77.8% of students preferred traditional classroom teaching compared to online learning, and almost one-third of students admitted to never attending online classes. 55.8% of students did not participate in pre-class preparation by reading e-books either before or after online classes [35]. Only 27% of students in Iraq found that the learning objectives of online learning were achieved and 67% of the same cohort reported fatigue or loss of interest associated with this new teaching method [41].

### Educator perspective

Students have not been the only population affected by the pandemic. A number of studies have also addressed concerns of educators in medical programmes. From these studies, medical teachers seem to be of a similar opinion in both HICs and LMICs, with the majority preferring face-to-face teaching with students. A survey among teachers from three universities in Paris found that 69.2% of teachers believed that they had not provided teaching to their students which was of an equivalent standard to that of pre-Covid times. Poor interaction and lack of feedback from students were reported as disadvantages [22]. In a South Korea university, 77.3% of teachers preferred traditional classroom education, while just 13.6% preferred online teaching. 9.1% reported having no preference between online and classroom teaching. However, 43.2% of teachers said that they hoped to maintain online teaching in combination with face-to-face lectures in the future and 4.5% wanted to continue online teaching only [20]. A survey of medical teaching staff in a South Indian university reported that all participants preferred regular classroom teaching, as it allowed better interaction with students. Nine of the 51 respondents felt the need to repeat the online classes in face-to-face teaching when it would be allowed to resume. Interestingly, one third of participants wanted the continuation of online teaching in addition to face-to-face teaching in the future [37].

### Technology and infrastructure

The use of technology and online learning platforms have been of paramount importance in delivering medical education during the pandemic and communicating with students while they are off-campus. The studies demonstrate that both HICs and LMICs experience some lack of access to digital resources or infrastructure required for effective e-learning. However, this disadvantage was significantly more prevalent in LMICs from the sample of studies included in this review. A survey of medical students in Nigeria demonstrated that both the educational faculties and students face serious technological burdens in this lower-middle-income country. Universities encountered challenges such as lack of online learning facilities and inadequate technical support while students faced the difficulty of not owning digital devices, the high cost of internet services and lack of power supply. This study reported that when all medical schools in Nigeria were forced to close due to the pandemic, only 45% of medical schools could continue to provide medical education online. In other universities, the transition to online teaching took up to eight weeks, resulting in students missing up to eight weeks of teaching in these cases [36]. Another study in the Philippines found that just 41% of medical students believed they could adapt to remote learning. Barriers to online learning included the absence of a fast and reliable internet connection and lack of physical space at home. One out of every ten students in this study reported always or often lacking basic requirements such as food, water, medicine and security [32]. Similar barriers were cited in other LMICs including China, India, Jordan, Libya and Sudan [29,30,34,37,43]. Technology and infrastructure issues were also reported in HICs, but to a lesser extent. A study at the University of California San Diego School of Medicine revealed that only 5.1% of students reported lack of sufficient internet or technology to access online resources [25]. A UK study found that 21.5% of medical students felt that internet connectivity was a barrier to e-learning. 11% reported that lack of space at home was an added burden [16].

### Curriculum and assessment

The pandemic has forced medical schools worldwide to deliver their curricula and assessments remotely to prevent the spread of COVID-19. A study in Jordan reported that only 26.8% of students felt satisfied with distance e-learning. An interesting point of this study was that using multimedia in the curriculum delivery improved satisfaction levels [29]. In France, approximately 15% of students and 38.5% of teachers were in favour of the use of an online curriculum after the pandemic [22]. Regarding assessment, a study in Egypt reported that 67.9% of medical students and educators used alternative methods for formative assessment but 75.6% reported a lack of alternative methods for summative examination. The approved summative assessment comprised of online projects. Some teaching staff felt this was a disappointing approach as summative exams were not included, which they believe motivate learning in students [38]. In Saudi Arabia, 66.9% of medical students said the pandemic had a positive effect on their education. However, 57.5% of students and educators in the same setting believed assessment was a challenge to online education [7]. A study from the University of Buffalo in the USA showed that 76% of medical students felt prepared for their examinations including the USA Medical Licensing Examination Step 2 clinical knowledge examination after the medical teaching they had received through online platforms [18]. 88% of students surveyed in a Pakistan study reported that they rejected online assessment as an alternative for traditional exams [31]. In the UK, 38.4% of final year medical students had their Objective Structured Clinical Examinations (OSCEs) cancelled, 18.6% had simulated patients or OSCE stations requiring patient contact cancelled while the remaining 43% had already completed their examinations before restrictions were imposed [5]. Similarly, in Australia, it was reported that campus-based examinations were instead held online in response to COVID-19 [26]. Interestingly, 66.4% of students in Germany were of the opinion that their teachers should be more lenient regarding examinations due to the disruption caused by COVID-19 [21].

## Discussion

This scoping review investigated the effect of the COVID-19 pandemic on medical education worldwide and how this effect differed in different income countries. From the results obtained, it is evident that COVID-19 has resulted in dramatic changes in the medical education structure globally. Medical education faculties in HICs and LMICs were placed under enormous pressure to deliver an online teaching platform amidst the emergency state caused by the pandemic. It is evident from the studies included in this review that this process was extremely challenging for students and educators in both HICs and LMICs. The majority of studies included in this review indicated that students from both cohorts reported that they did not feel that the education they were receiving through e-learning matched the standard of teaching that they would have received in normal pre-COVID times [4,5,16,41]. Students reported missing out on practical elements of their education and felt this would impact negatively on their careers [16,24,25]. Students from both HICs and LMICs also reported poorer mental health due to the effect of COVID-19 on their education. Reasons for this included worrying about the effect of the pandemic on their future career prospects, lack of motivation to study, loneliness and isolation [18,21,23,31,32]. Students were not the only population affected by these radical changes in medical education. Educators also expressed frustration at online learning. The studies indicated that the majority of teachers prefer traditional face-to-face teaching for a variety of reasons including a better standard of interaction with students and ability to receive more feedback [20,22,37].

Pre-Covid times, e-learning was already used to complement traditional face-to-face medical education in some universities [44]. Vaona *et al*. concluded that ‘when compared to traditional learning, e-learning may make little or no difference in patient outcomes or health professionals’ behaviours, skills or knowledge.’ However, there are also barriers associated with e-learning, which were outlined in some of the studies included in this review. Insufficient information technology (IT) infrastructure, lack of internet supply and lack of staff training were some of the challenges outlined by both students and educators in HICs and LMICs [13,22,27,29,35,36,43]. Positive aspects of the new online platforms were also reported. Medical students in both HICs and LMICs enjoyed the increased flexibility of not physically attending classes. Time and financial savings were also included as benefits of remote learning [16,33,34]. However, it was evident from the studies included in this review that students and educators did not view a full online curriculum as a satisfactory alternative to traditional face-to-face teaching and the practical experience of clinical placements [13,35,40]. Many studies from HICs described the favouring of a hybrid teaching approach in the future, meaning that both face-to-face teaching and online teaching could be used in combination to provide a greater standard of education [15,17,20,28]. Interestingly, the attitude towards online learning in the future was less discussed in studies from LMICs.

There were many similarities in the results obtained from studies in HICs and LMICs. It was interesting to see that worldwide, many of the same advantages and challenges arose for medical schools and their students and teachers. However, the severity of disadvantages experienced in LMICs due to lack of resources appeared to be far greater than that experienced in HICs. For example, in Nigeria, students were unable to transition to online learning for up to eight weeks in some cases [36]. This type of delay in providing an online platform was not reported in any HIC. Overall, disadvantages such as lack of infrastructure, poor internet connectivity and lack of space at home were reported to a greater extent in the studies obtained from LMICs. This result is expected as LMICs have less financial resources to cope with the challenges posed by this pandemic. An example which highlights the barriers faced by LMICs is that from March 2020 until January 2021, all public universities in Nigeria remained closed as they could not cope with the devastation caused by COVID-19 and social distancing could not be facilitated in these universities. Therefore, there were no medical graduates from these schools during that time period and students faced severe disruption to their education [2]. Lack of personal protective equipment was also reported as a significant worrying factor for students on clinical placement in LMICs [2].

This scoping review has identified gaps in knowledge regarding this topic. While the review has aided in identifying some of the effects of COVID-19 on medical education, it has not presented solutions or methods of dealing with these impacts. Future studies should be directed at addressing the barriers and challenges encountered by medical schools in providing education during a pandemic. It cannot be known if medical education will ever return to the way it was in pre-Covid times, and therefore measures should be taken to ensure that this education can be executed in the most effective way possible in all countries. The healthcare systems of LMICs are already under pressure, and education of medical students is necessary for systems to cope. The World Health Organisation (WHO) reported that in 2013, the world’s poorest regions, Africa and South-East Asia had the lowest numbers of doctors (0.3 and 0.6 doctors per 1000 population respectively) but had the greatest global burdens of preventable disease [45]. It is likely that these burdens will intensify if these issues are not addressed. In addition, future research should discover the benefits of the changes made to medical education as a result of COVID-19. In this way, positive aspects of these changes can be incorporated into the future medical education structure. This scoping review also reflected that there is an evidence gap regarding how medical students have performed in their examinations following exposure to the changes caused by COVID-19. It would be interesting to quantify how these changes have impacted on students’ academic performance.

There were several strengths and limitations associated with this scoping review. In relation to strengths, this review strictly followed an effective methodology design by Arksey and O’Malley. A constructive study selection process was also used, which ensured the studies included were relevant to the research question. The findings of this scoping review are in relation to a new subject and one that there is still very little research into thus far. Therefore, these results provide a valuable foundation for future studies. There were also limitations encountered. The heterogeneity of the studies included in this review means that it is difficult to form a single conclusion from the data provided. The outcomes obtained from the articles were broad, and many did not answer simply a single research question, but they encompassed much information regarding important topics related to the central issue of how COVID-19 has impacted medical education. Therefore, a broad overview of the literature available was presented in this review rather than a focused, single answer to the research question. However, heterogeneity is a common result of medical education literature reviews [46]. Although every effort was made to ensure these results were robust and justified, it cannot be ruled out that they may be affected by factors such as bias. This is a scoping review, which means that the studies included did not undergo quality appraisal processes. The nature of scoping reviews means that multiple publication forms can be included. Arksey and O’Malley emphasise that comprehensiveness is a key aspect of scoping reviews [10]. However, this means that the quality standard of studies included in this review did not undergo rigorous evaluation. It must also be noted that the review was limited to articles written in the English language. Studies published in other languages were not included, and this may have introduced bias. Finally, this review examined the effect of COVID-19 on undergraduate medical education only. It did not include other healthcare team students or medical professionals. A study encompassing a broader population may be useful in the future to give an insight into the effects of the pandemic on other healthcare students and professionals.

## Conclusions

This review has highlighted the wide range of barriers encountered by medical schools throughout the world in response to COVID-19. The main issues reported by medical students and educators include insufficient IT infrastructure, lack of patient contact, lack of trained personnel and mental health burdens. The review also assessed these factors in relation to different income countries and investigated similarities and differences. LMICs face unique challenges due to limited resources, and therefore adapting their curricula to the pandemic has proved to be very difficult. This review demonstrated that there are also positive aspects associated with remote learning, and these should be taken into account when planning future teaching. Despite the chaos caused by the pandemic, there have been valuable lessons learned, and these should not be ignored. Students’ and educators’ opinions regarding the greater flexibility and benefits associated with remote learning should be strategically considered for future curricula. The outcome of COVID-19 on medical education is still unknown, and therefore action must be taken to prepare for any eventuality.
